# A non-invasive continuous and real-time volumetric monitoring in spontaneous breathing subjects based on bioimpedance—ExSpiron®Xi: a validation study in healthy volunteers

**DOI:** 10.1007/s10877-023-01107-0

**Published:** 2024-01-19

**Authors:** Stefano Gatti, Emanuele Rezoagli, Fabiana Madotto, Giuseppe Foti, Giacomo Bellani

**Affiliations:** 1 Department of Emergency and Intensive Care, Terapia Intensiva e Semintensiva adulti e Pediatrica, Fondazione IRCCS San Gerardo dei Tintori, Monza, Italy; 2 School of Medicine and Surgery, University of Milano-Bicocca, Monza, Italy; 3 Department of Area Emergenza Urgenza, Fondazione IRCCS Ca’ Granda - Ospedale Maggiore Policlinico, Milan, Italy; 4 Centre for Medical Sciences - CISMed, University of Trento, Trento, Italy; 5 Anesthesia and Intensive Care, Santa Chiara Regional Hospital, APSS Trento Largo Medaglie d’Oro, Trento, Italy

**Keywords:** Tidal volume, Bioimpedance, Non-invasive monitoring, Respiratory failure, Healthy volunteers, Non-invasive ventilation, Non-invasive respiratory support

## Abstract

Tidal volume (TV) monitoring breath-by-breath is not available at bedside in non-intubated patients. However, TV monitoring may be useful to evaluate the work of breathing. A non-invasive device based on bioimpedance provides continuous and real-time volumetric tidal estimation during spontaneous breathing. We performed a prospective study in healthy volunteers aimed at evaluating the accuracy, the precision and the trending ability of measurements of ExSpiron®Xi as compared with the gold standard (i.e. spirometry). Further, we explored whether the differences between the 2 devices would be improved by the calibration of ExSpiron®Xi with a pre-determined tidal volume. Analysis accounted for the repeated nature of measurements within each subject. We enrolled 13 healthy volunteers, including 5 men and 8 women. Tidal volume, TV/ideal body weight (IBW) and respiratory rate (RR) measured with spirometer (TV_Spirometer_) and with ExSpiron®Xi (TV_ExSpiron_) showed a robust correlation, while minute ventilation (MV) showed a weak correlation, in both non/calibrated and calibrated steps. The analysis of the agreement showed that non-calibrated TV_ExSpiron_ underestimated TV_spirometer_, while in the calibrated steps, TV_ExSpiron_ overestimated TV_spirometer_. The calibration procedure did not reduce the average absolute difference (error) between TV_Spirometer_ and TV_ExSpiron_. This happened similarly for TV/IBW and MV, while RR showed high accuracy and precision. The trending ability was excellent for TV, TV/IBW and RR. The concordance rate (CR) was >95% in both calibrated and non-calibrated measurements. The trending ability of minute ventilation was limited. Absolute error for both calibrated and not calibrated values of TV, TV/IBW and MV accounting for repeated measurements was variably associated with BMI, height and smoking status. Conclusions: Non-invasive TV, TV/IBW and RR estimation by ExSpiron®Xi was strongly correlated with tidal ventilation according to the gold standard spirometer technique. This data was not confirmed for MV. The calibration of the device did not improve its performance. Although the accuracy of ExSpiron®Xi was mild and the precision was limited for TV, TV/IBW and MV, the trending ability of the device was strong specifically for TV, TV/IBW and RR. This makes ExSpiron®Xi a non-invasive monitoring system that may detect real-time tidal volume ventilation changes and then suggest the need to better optimize the patient ventilatory support.

## Introduction

Elevated tidal volume (TV), especially if leading to elevated driving and plateau pressure, is widely accepted as one of the major determinants of ventilator induced lung injury (VILI) in mechanically ventilated subject with respiratory failure [1]. For this reason, the use of protective mechanical ventilation, with limitation of TV and inspiratory pressures has become the standard of care in patients with acute respiratory distress syndrome (ARDS) [2]. Recently, along with an increased use of non-invasive ventilatory support strategies, the risk of patient self-inflicted lung injury (P-SILI) is increasingly recognized in patients with acute respiratory failure [3]. In this context patients can generate injurious TV, while the potential for clinicians to take control over these is limited. As an example, Carteaux et al. showed that TV is independently associated with the risk of non-invasive ventilation (NIV) failure in de-novo respiratory failure [4].

Consequently, a potential monitoring tool of TV and minute ventilation (MV) would be of high clinical relevance to ensure safety and optimize the management of NIV in spontaneous breathing subjects, also as a tool to support the decision of proceeding to intubation. However, TV monitoring is hardly achieved in non-intubated patients, as opposed to the setting of invasive ventilation, and this was clearly evident during the recent COVID pandemic [5] especially in the out of ICU clinical enviroments [6]. While in patients undergoing NIV via face mask the mechanical ventilator quantifies a TV (albeit these still bear relevant limitations, deriving from the volume of compression of the mask and the leaks), this is not the case for other devices which are rapidly gaining popularity, such as head helmets [7, 8] and high-flow nasal cannula HFNC [9].

ExSpiron®Xi is a noninvasive monitor that was developed to provide a continuous real-time measurement of TV, respiratory rate (RR) and MV, based on bio-impedance technology through a set of electrodes placed on the subjects’ chest. Clinical studies were performed in order to investigate the usefulness of this technique, as compared to standard of care in various settings such as procedural sedations [10] and perioperative care [11, 12].

Since only one study evaluated the agreement between bioimpedance and standard [13], in this physiological study we aimed at evaluating the correlation between ExSpiron®Xi measurements in healthy volunteers and spirometry ones—as a standard technique to evaluate tidal volume ventilation. We further aim at assessing the accuracy of measurements of ExSpiron®Xi in healthy volunteers as compared to spirometry and we sought to determine if the accuracy would be improved by the calibration with a pre-determined tidal volume obtained through a non-expandable bag in analogy with previous application [14]. At last, we aimed at determining the trending ability of the change in tidal volume between ExSpiron®Xi and the standard technique. We used different combinations of tidal volume and respiratory rate to mimic a wide range of potential clinical scenarios.

## Materials and methods

This is a prospective study performed in healthy volunteers from August to October 2021. The study was approved by the Ethics Committee of the University of Milano Bicocca (Prot. 0071913/21 on June 11th 2021), and all the subjects provided informed consent.

All subjects in good health and without ongoing acute or chronic pulmonary diseases were considered eligible. Age under 18 years old, pregnancy and contraindications to electrical impedance use were considered exclusion criteria.

Demographic (i.e. age and sex), smoking habit, and anthropometric information (i.e. weight, height and body mass index—BMI) were collected at the time of enrollment.

### Study protocol

Subjects were seated on a chair and were connected to ExSpiron®Xi (Respiratory Motion Inc., Watertown, MA) through a disposable single-use padset composed by three electrodes applied on the thorax, according to manufacturer recommendations. One electrode was placed under the suprasternal notch, the second one under the xiphoid and the third one on the right midaxillary line at the level of the xiphoid. Continuous and real-time data of TV, RR and MV measured by ExSpiron®Xi, were downloaded from the instrument and off-line analyzed.

Subjects were asked to breathe through a mouthpiece connected to a previously calibrated spirometer wearing a nose clip. A single-use mouthpiece with High Efficiency Particulate Air (HEPA) filter was applied. Spirometry values were continuously recorded by LabChart® software (ADInstruments, Dunedin, New Zealand). Volume was obtained by flow integration and drift corrected by resetting zero at each inspiration**.**

Ideal body weight (IBW) of the subjects was calculated as: 50 + 0.91(centimeters of height − 152.4) for male volunteers and 45.5 + 0.91(centimeters of height − 152.4) for female [1]. Five target tidal volumes were calculated multiplying the ideal body weight of each subject by 4, 8, 12, 16 and 20 mL/kg IBW.

The study was divided into two phases.

### First phase

This phase included five steps, during which subjects were asked to breathe regularly achieving a target TV (4, 8, 12, 16 or 20 mL/kg IBW) in random order. In order to achieve this, the actual TV waveform, superimposed on the target TV were continuously shown to each subject in order to provide a visual feedback. After target was reached and shortly maintained, the actual recording begun, lasting 90 s, followed by a period of rest before the start of the following step.

### Second phase

Since the ExSpiron®Xi foresees the possibility of calibration with a known tidal volume (e.g. in intubated subjects before extubation), we sought a simple way to obtain a “standard” TV, possibly applicable in daily clinical practice. Hence subjects were asked to completely expand and collapse a non-elastic calibration bag made with a known volume of 1300 mL (Flow-meter, Levate, Italy) placed after the spirometer. They breathed five times distending completely the balloon at the end of expiration and collapsing it at the end of inspiration. During this period ExSpiron®Xi was set on calibration mode using the device function. Once the calibration was completed, the subjects were asked to repeat the five TV steps (4, 8, 12, 16 or 20 mL/kg IBW) as previously described in first phase.

### Statistical analysis

Continuous data were reported as mean ± standard deviation (SD). Data distributions were assessed for normality by visual inspection.

For each parameter measured by spirometry and by ExSpiron®Xi, we performed a scatter plot considering all target of TV (4, 8, 12, 16, 20 mL/Kg IBW). Relationship between the two measurements was evaluated by the repeated measures correlation coefficient (r_m_) for determining the overall within-individual relationship among paired measures [15]. Differences between devices at each target of TV were statistically tested using generalized mixed linear regression model.

The agreement between spirometry and ExSpiron®Xi data, both in their calibrated and non-calibrated values, was assessed using the Bland–Altman method, enabling us to estimate any biases present and determine the limits of agreement (LoA). Limits of agreements and 95% confidence interval were estimated accounting for the longitudinal and repeated nature of measurements within each subject. For each parameter, by dividing the limit of agreement thus calculated by the mean, we estimated the percentage error (PE). A PE of < 30% is suggested to be acceptable for clinical use [16]. Moreover, the concordance correlation coefficient (CCC) accounting for repeated measures per subject was calculated [17].

For each parameter, to evaluate the ability to track changes (trending ability) of the device, we performed a 4-quadrant plot analysis, reporting all differences between measurements obtained with spirometry and ExSpiron®Xi [16]. We calculated the Concordance Rate (CR) by determining the proportion of data points in which both devices exhibited changes in the same direction, as depicted in the blue area of the plot. 95% confidence interval (CI) of CR was estimated using the exact method based on binomial distribution.

To also investigate the relationship between the absolute differences (our dependent variable) and each individual’s demographic and anthropometric parameter (independent variable), we performed a generalized mixed linear regression model, considering the issue of multiple measurements per subject.

All statistical tests were two-sided and assumed a significance α-level of 0.05. Analyses were performed using R 4.3.1 (R Foundation for Statistical Computing, Vienna, Austria).

## Results

### Study population

We enrolled 13 healthy volunteers, including 5 men and 8 women; 5 of them were active smokers. Mean age was 35.2 ± 11.9 years, weight 67.2 ± 18.4 kg, height 1.71 ± 0.14 m and BMI 22.6 ± 3.0 kg/m^2^.

### Concordance between estimated and measured tidal volume, respiratory rate and minute ventilation

**Fig. 1:**
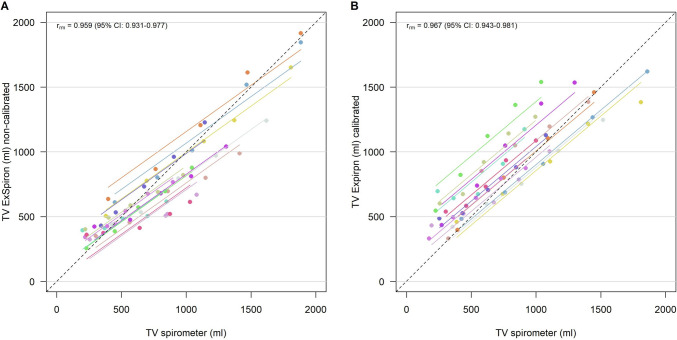
Scatter plot of **A** TV_ExSpiron_ and **B** TV_spirometer_ in non-calibrated and calibrated steps. *TV* tidal volume, *r*_*m*_ repeated measures correlation coefficient

TV_spirometer_ and TV_ExSpiron_ showed a strong correlation both before (r_rm_ = 0.959—95% CI 0.931–0.977) and after (r_rm_ = 0.967—95% CI 0.943–0.981) calibration, Fig. 1.

**Fig. 2:**
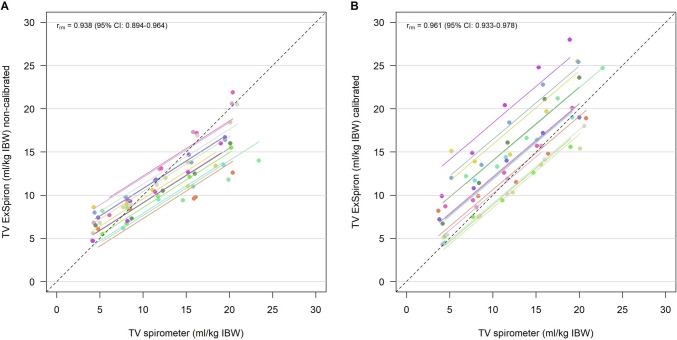
Scatter plot of **A** TV/IBW_ExSpiron_ and **B** TV/IBW_spirometer_ in non-calibrated and calibrated steps. *TV* tidal volume, *IBW* ideal body weight, *r*_*m*_ repeated measures correlation coefficient

**Fig. 3:**
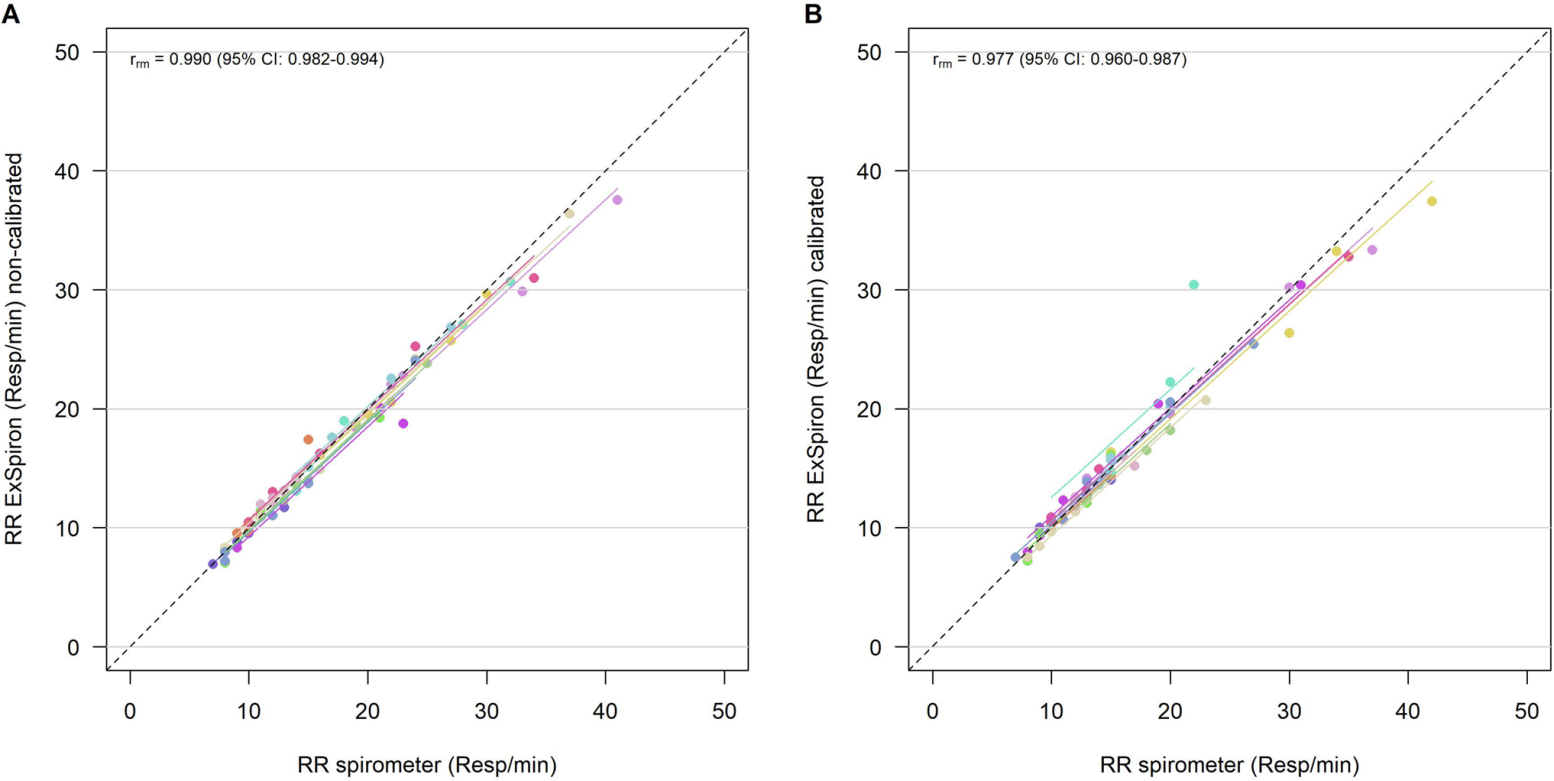
Scatter plot of **A** RR_ExSpiron_ and **B** RR_spirometer_ in non-calibrated and calibrated steps. *RR* respiratory rate, *r*_*m*_ repeated measures correlation coefficient

**Fig. 4:**
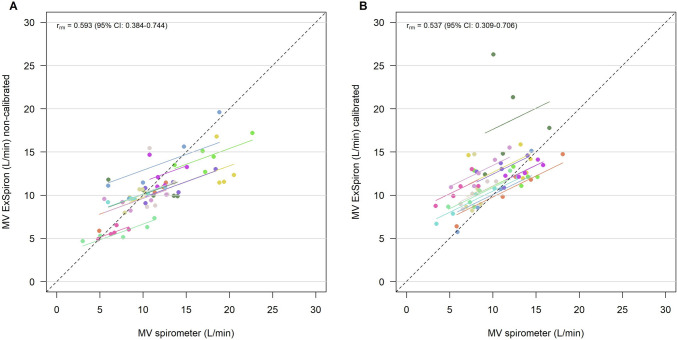
Scatter plot of **A** MV_ExSpiron_ and **B** MV_spirometer_ in non-calibrated and calibrated steps. *MV* minute ventilation, *r*_*m*_ repeated measures correlation coefficient

We observed a robust strong correlation in TV/IBW between the techniques before (r_rm_ = 0.938—95% CI 0.894–0.964) and after (r_rm_ = 0.961—95% CI 0.933–0.978) calibration, Fig. 2. RR_spirometer_ and RR_ExSpiron_ showed excellent correlation both before (r_rm_ = 0.990—95% CI 0.982–0.994) and after calibration (r_rm_ = 0.977—95% CI 0.960–0.987), Fig. 3. We observed a weak correlation between MV measurements obtained in non-calibrated (r_rm_ = 0.593—95% CI 0.384–0.744) and calibrated (r_rm_ = 0.537—95% CI 0.309–0.706) steps, Fig. 4.

**Fig. 5:**
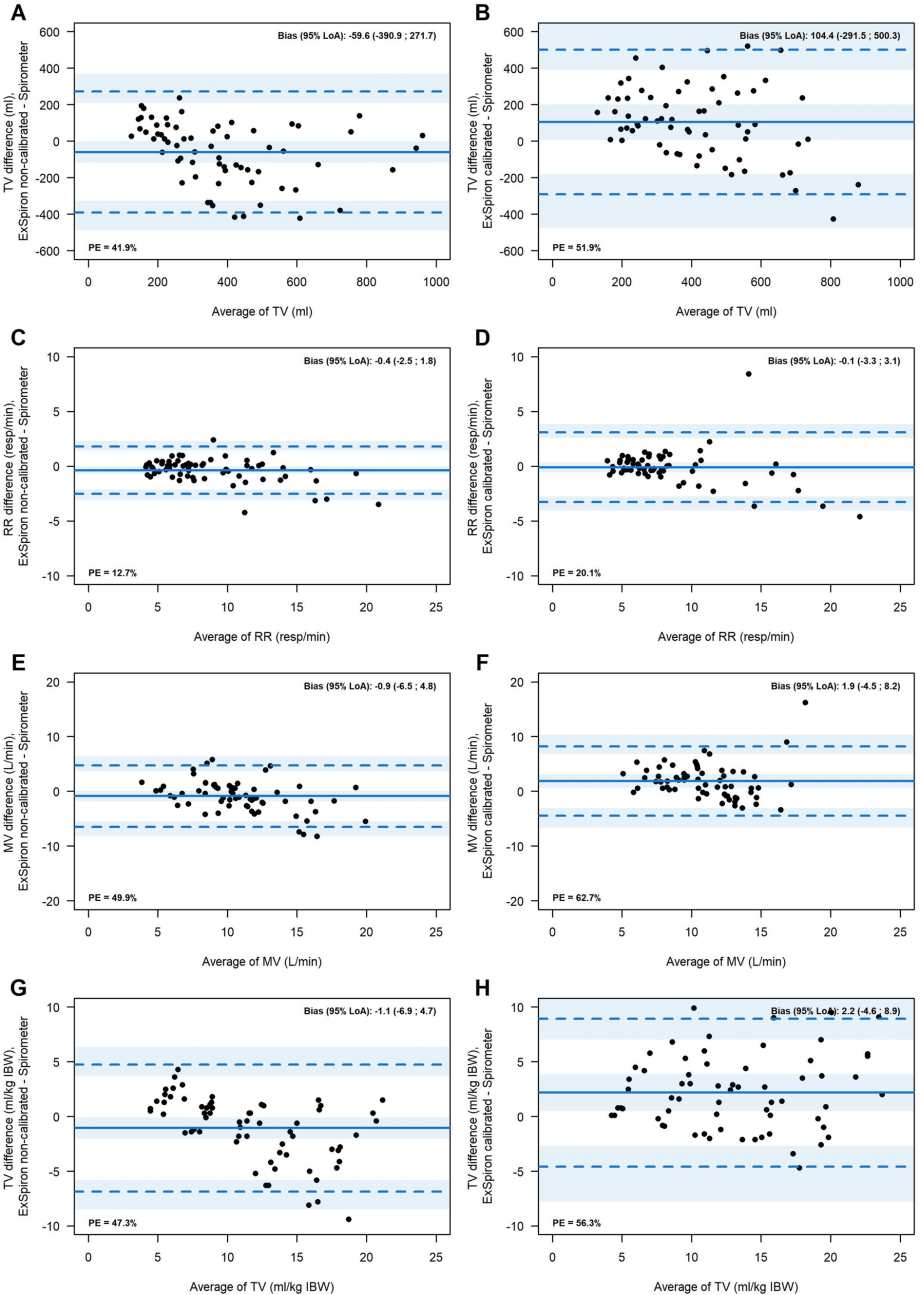
Bland-Altam plots for each parameter estimated by ExSpiron and measured by spirometry. Tidal volume (TV) in non-calibrated (**A**) and calibrated (**B**) steps. Respiratory rate (RR) in non-calibrated (**C**) and calibrated (**D**) steps. Minute ventilation (MV) in non-calibrated (**E**) and calibrated (**F**) steps. TV/ideal body weight (TV/IBW) in non-calibrated (**G**) and calibrated (**H**) steps. In each plot percentage error (PE) is reported. LoA limits of agreement

The analysis of the agreement (Fig. 5—Panel A and B) showed that non-calibrated TV_ExSpiron_ underestimated TV_spirometer_ by 59.6 mL 95% LoA [− 390.9, 271.7], while in the calibrated steps, TV_ExSpiron_ overestimated TV_spirometer_ of 104.4 mL 95% LoA [− 291.5, 500.3]. Percentage error was 41.9% in non-calibrated steps and 51.9% in calibrated ones.

RR showed a high agreement in both non calibrated and calibrated steps between ExSpiron®Xi and the spirometer (Fig. 5—Panel C and D), in particular, non-calibrated RR_ExSpiron_ underestimated RR_spirometer_ by − 0.4 resp/min 95% LoA [− 2.5, 1.8], while in the calibrated steps, RR_ExSpiron_ underestimated RR_spirometer_ of − 0.1 resp/min 95% LoA [− 3.3, 3.1]. Percentage error was 12.7% in non-calibrated steps and 20.1% in calibrated ones.

Non-calibrated MV_ExSpiron_ underestimated MV_spirometer_ by 0.9 L/min 95% LoA [− 6.5, 4.8], while in the calibrated steps, MV_ExSpiron_ overestimated MV_spirometer_ of 1.9 L/min 95% LoA [− 4.5, 8.2], Fig. 5—Panel E and F. Percentage error was 49.9% in non-calibrated steps and 62.7% in calibrated ones.

Non-calibrated TV/kgIBW _ExSpiron_ underestimated TV/kgIBW _spirometer_ by 1.1 mL/kg 95% LoA [− 6.9, 4.7], while in the calibrated steps, TV/kgIBW _ExSpiron_ overestimated TV/kgIBW _spirometer_ of 2.2 mL/kg 95% LoA [− 4.6, 8.9], Fig. 5—Panel G and H. Percentage error was 47.3% in non-calibrated steps and 56.3% in calibrated ones.

### Differences between ExSpiron®Xi and spirometer over increasing levels of TV/IBW

**Fig. 6:**
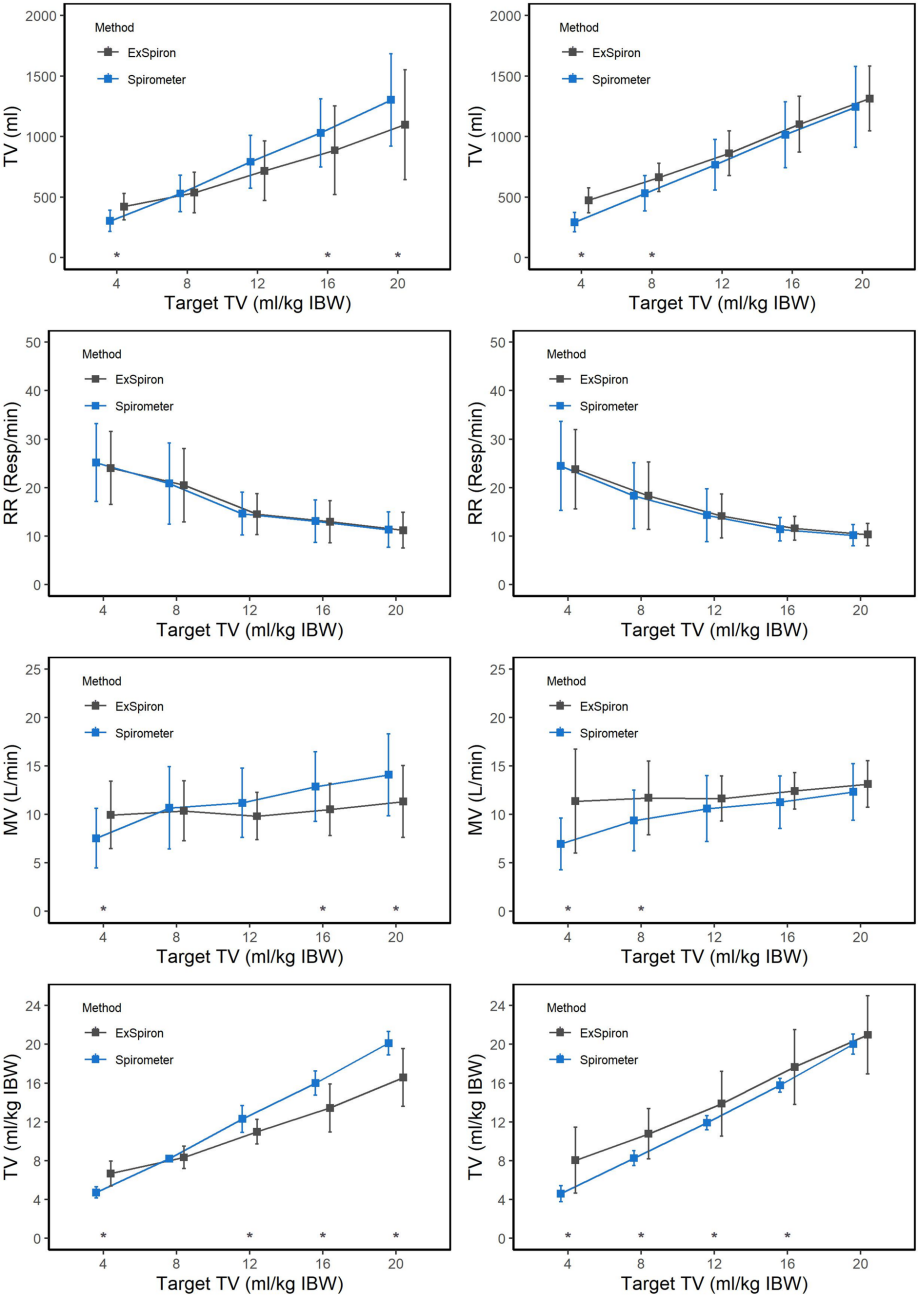
2-way ANOVA differences for each parameter measured by spirometry and by ExSpiron®Xi over increasing tidal volume. On the left are reported graphs for non-calibrated steps, on the right for calibrated. *, p-value<0.05 versus spirometer at a specific target TV. *TV* tidal volume, *RR* respiratory rate, *MV* minute ventilation, *IBW* ideal body weight

The devices differed in TV, MV and TV/IBW in both non-calibrated and calibrated steps. Interestingly, ExSpiron®Xi overestimated TV, MV and TV/IBW at lower values while it down estimated the same variables at higher values in the non-calibrated steps as compared to Spirometer. Contrarily, ExSpiro®Xin always overestimated TV, MV and TV/IBW and these differences were statistically significant mainly at lower levels of TV/IBW as compared to Spirometer (Fig. 6).

### Factors influencing the differences between measured parameters

In Table 1 is reported the relationship between the absolute differences of the measured parameters and demographic-anthropometric features of the healthy subjects. In non-calibrated steps, TV difference was statistically associated to smoking habit, TV/IBW difference was statistically associated to smoking habit and height. In calibrated steps, TV an TV/IBW differences were both associated significantly to BMI and TV/IBW difference was significantly associated with sex. MV difference was significantly associated to height.

**Table 1:** Relationship between the absolute differences and demographic-anthropometric parameters

Dependent variable	Independent variable	Non-calibrated		Calibrated	
		Beta ± SE	p value	Beta ± SE	p-value
Absolute differences TV (mL)	Age (1 year increase)	0.08 ± 1.56	0.9581	− 0.49 ± 2.82	0.8656
	Sex (Ref. Female)	1.01 ± 36.65	0.9786	94.64 ± 59.96	0.1427
	Height (1 meter increase)	− 82.83 ± 132.00	0.5430	− 258.40 ± 230.90	0.2866
	BMI (1 kg/m2 increase)	1.26 ± 6.10	0.8406	− 24.57 ± 8.19	**0.0121**
	Smoke (Ref. No)	69.84 ± 30.00	**0.0400**	63.28 ± 63.49	0.3405
Absolute differences RR (resp/min)	Age (1 year increase)	− 0.01 ± 0.01	0.3277	0.01 ± 0.01	0.4044
	Sex (Ref. Female)	0.05 ± 0.22	0.8232	0.09 ± 0.35	0.8061
	Height (1 meter increase)	0.03 ± 0.81	0.9730	− 0.46 ± 1.30	0.7316
	BMI (1 kg/m2 increase)	0.04 ± 0.04	0.2294	0.01 ± 0.06	0.8192
	Smoke (Ref. No)	0.21 ± 0.22	0.3482	0.20 ± 0.35	0.5688
Absolute differences MV (L/min)	Age (1 year increase)	− 0.01 ± 0.03	0.6627	− 0.03 ± 0.04	0.4195
	Sex (Ref. Female)	− 0.22 ± 0.73	0.7667	− 1.65 ± 0.84	0.0740
	Height (1 meter increase)	− 1.63 ± 2.64	0.5496	− 6.93 ± 2.93	**0.0365**
	BMI (1 kg/m2 increase)	− 0.03 ± 0.12	0.8162	− 0.26 ± 0.14	0.1008
	Smoke (Ref. No)	1.05 ± 0.66	0.1405	1.50 ± 0.86	0.1095
Absolute differences TV (mL/kg IBW)	Age (1 year increase)	0.02 ± 0.03	0.5477	− 0.01 ± 0.06	0.8408
	Sex (Ref. Female)	− 0.99 ± 0.72	0.1971	− 2.69 ± 1.06	**0.0280**
	Height (1 meter increase)	− 5.33 ± 2.37	**0.0457**	8.81 ± 4.12	0.0556
	BMI (1 kg/m2 increase)	− 0.13 ± 0.12	0.3240	− 0.56 ± 0.15	**0.0027**
	Smoke (Ref. No)	1.82 ± 0.55	**0.0071**	1.75 ± 1.23	0.1826

Results from univariate generalized mixed linear models accounting for repeated measures

Bold in Table 1 means that p-value<0.05 therefore it is statistically significant

*BMI* body mass index, *IBW* ideal body weight, *MV* minute ventilation, *RR* respiratory rate, *SE* standard error, *TV* tidal volume

### Trending ability and concordance rate

**Fig. 7:**
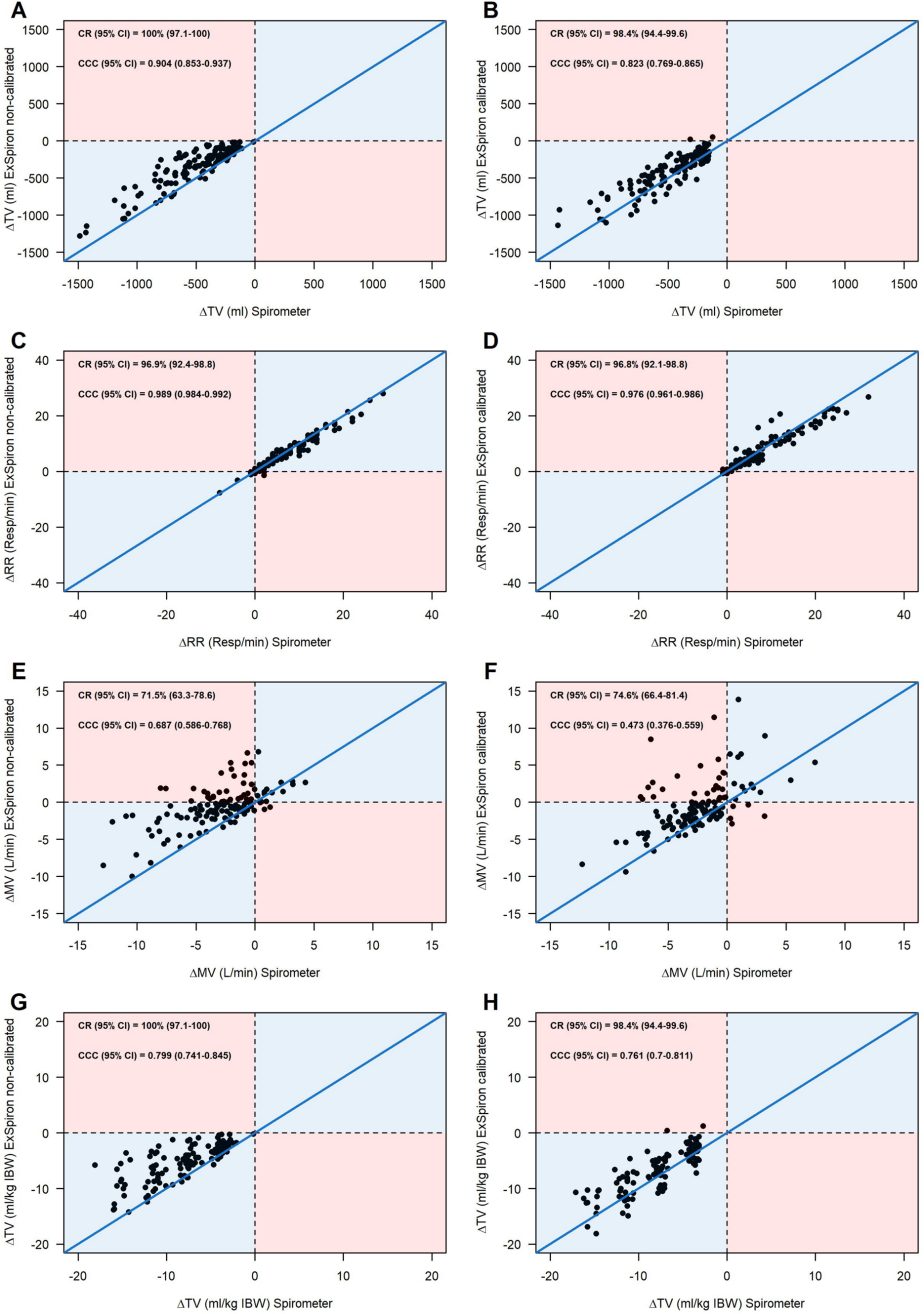
4-quadrant plots reporting trending ability for each parameter estimated by ExSpiron and measured by spirometry. Change in tidal volume (ΔTV) in non-calibrated (**A**) and calibrated (**B**) steps. Change in respiratory rate (ΔRR) in non-calibrated (**C**) and calibrated (**D**) steps. Change in minute ventilation (ΔMV) in non-calibrated (**E**) and calibrated (**F**) steps. Change in TV/ideal body weight (ΔTV/IBW) in non-calibrated (**G**) and calibrated (**H**) steps. In each plot concordance rate (CR) and concordance correlation coefficient (CCC) are reported

The trending ability of ExSpiron®Xi is expressed by the 4-quadrant plots reported in Fig. 7. The concordance correlation coefficient (CCC) between ΔTV spirometer and ΔTV ExSpiron®Xi was 0.904 95% CI [0.853;0.937] in non-calibrated measurements and 0.823 95% CI [0.769;0.865], concordance rate (CR) was 100% 95%CI [97.1;100] for non-calibrated measurements and 98.4% 95%CI [94.4;99.6] for calibrated ones, Fig. 7—Panel A and B. Similar findings were oserved in the 4-quadrant plots of RR and TV/kgIBW that are reported in Fig. 7—Panel C, D, G and H, CCC and CR are reported in the graphs.

Concerning MV (Fig. 7—Panel E and F), concordance correlation coefficient (CCC) between ΔMV spirometer and ΔMV ExSpiron®Xi was 0.687 95% CI [0.586;0.768] in non-calibrated measurements and 0.473 95% CI [0.376;0.559], concordance rate (CR) was 71.5% 95%CI [63.3;78.6] for non-calibrated measurements and 74.6% 95%CI [66.4;81.4] for calibrated ones.

## Discussion

In this prospective physiological study performed in healthy volunteers we obtained the following main results:

1. The correlation of TV, TV/IBW and RR estimated with the ExSpiron®Xi device as compared with the gold standard spirometer was robust; contrarily the correlation in minute ventilation between ExSpiron®Xi device and the gold standard spirometer was weak in both calibrated and non-calibrated steps;
2. Tidal volume, TV/IBW and MV are underestimated at high tidal volumes and overestimated at low tidal volumes with ExSpiron®Xi in non-calibrated steps and are overestimated in the calibrated ones, as compared with the spirometer analysis. Accuracy of ExSpiron®Xi in estimating TV, TV/IBW and MV by the Bland–Altman analyses is low, the limits of agreements are wide and the percentage error is elevated both in non-calibrated and calibrated steps;
3. ExSpiron®Xi has an excellent trending ability on the detection of TV, TV/IBW and RR change when compared with the spirometer, however it has a low trending ability in detecting MV.

The detection of absolute values of tidal volumes and TV/IBW with ExSpiron®Xi compared with spirometer shows a robust correlation in non-calibrated and calibrated steps. Regarding the respiratory rate, the correlation between ExSpiron®Xi and spirometer is excellent as expected considering the known accurate measure of this parameter based on bioimpedance [18]. However, the correlation between the tested device on minute ventilation is weak. This might be explained because 1. ExSpiron®Xi overestimates TV spirometer data at low TV values while it underestimates spirometer data at high TV levels in the non-calibrated steps, and 2. because ExSpiron®Xi does not provide a breath-by-breath data analysis and a certain delay is present [13, 19].

The accuracy of ExSpiron®Xi is limited. The study device underestimates TV, TV/IBW and MV in non-calibrated measurements while it overestimates it in calibrated ones. Moreover, as shown by Bland–Altman plots, precision of ExSpiron®Xi is poor, as shown by the wide range of 95% CI around the bias.

The performance of ExSpiron®Xi in detecting variation of TV and TV/IBW across different steps is excellent. As a matter of fact, ExSpiron®Xi has an excellent trending ability regarding tidal volume when compared to the gold standard technique. Therefore, ExSpiron®Xi can detect changes in tidal volume in spontaneously breathing subjects regardless the calibration. In contrast, trending ability of minute ventilation is poor. Respiratory rate estimated by ExSpiron®Xi is the parameter that matches all the excellent requirements of correlation, accuracy, precision and treanding ability as compared with the spirometer.

The comparison of impedance-based Respiratory Volume Monitor such as ExSpiron®Xi and other reference methods was reported in the literature [19–22], however, a few studies compared the performance of ExSpiron®Xi with the gold standard technique of tidal volume measurement.

Our findings reported a lower ExSpiron®Xi performance as compared with the data reported by Voscopoulos et al. [13]. This is probably the consequence of different study settings: while Voscopoulos et al. investigated a variety of different respiratory patterns by mainly modulating RRs, in our study we explored the change in respiratory patterns by exploring changes in tidal volumes ranging from very low tidal volume ventilation (4 mL/kg of IBW) up to 20 mL/kg of IBW.

Although through a preliminary analysis, we observed that demographic data may play a role in the different detection of tidal volume ventilation by ExSpiron®Xi as compared with spirometer. These include a different BMI and a smoking habit, that may have an impact on the quantification of tidal volume because of atelaectasis and airway closure [23]—the first—and slow time of exhalation [24]—the second.

Tidal volume during spontaneous breathing is increasingly recognized as a key parameter in leading to patient-self inflicted lung injury (P-SILI) [3]. Carteaux et al. showed that increasing tidal volumes during NIV may predict intubation [3, 4]. Frat et al. highlighted how patients who failed NIV have high tidal volumes before intubation [3, 25]. Therefore, it ExSpiron®Xi may provide the opportunity to monitor tidal volume in real time in patients with respiratory failure during spontaneous breathing, allowing to potentially prevent P-SILI and to plan an early intubation. So far, it is not possible to monitor tidal volume during spontaneous breathing during non-invasive respiratory support—with the exception face-mask NIV connected to a ventilator. The use of ExSpiron®Xi may allow the measurement of tidal volume in real-time in non-intubated patients. ExSpiron®Xi measurements have a low accuracy but low precision. However, the excellent ability of the instrument to detect positive and negative variation of the tidal volume over time (i.e. trending ability) may be of high clinical relevance for the physicians. This may allow to better understand improvements or deteriorations in patients’ respiratory patterns and then suggest an earlier change in the respiratory support strategy—non-invasive or invasive. Consequentially, the study device ensure safety and optimize the respiratory management in spontaneously breathing patients. Furthermore, ExSpiron®Xi is a non-invasive monitoring device that makes its use feasible handy at bedside in patients who are not intubated and who do not have a real time continuous monitoring system of tidal volume and minute ventilation as in the case of invasive mechanical ventilation [6].

The possibility to track intrathoracic volume changes by means of bioimpedance change is by using some devices [14, 26]. However, to the best of our knowledge, the ExSpiron®Xi monitoring tool is the only one providing levels of absolute tidal volumes.

We acknowledge that we investigated the performance of ExSpiron®Xi in estimating tidal volume ventilation in healthy volunteers who performed experiments in a sitting position to strictly follow the study protocol. It is well known how different positions may affect respiratory mechanics, in particular in pathological settings [27, 28]. However, in this study our aim was to investigate the performance of ExSpiron®Xi in a physiological setting.

In conclusion, non-invasive tidal volume estimation by ExSpiron®Xi was strongly correlated with the gold standard spirometer technique. The performance of ExSpiron®Xi did not improve after device calibration. Although the accuracy of ExSpiron®Xi was mild and the precision was limited, the trending ability of the device was excellent for individual measurements of tidal volume and respiratory rate. The reliability of the ExSpiron®Xi device to estimate the TV, TV/IBW and RR in spontaneously breathing subjects—non-invasively—is of potentially great clinical relevance in the setting of respiratory failure. The robust trending ability of ExSpiron®Xi makes this device of potential clinical use at bedside to detect harmful respiratory patters such as high tidal volume ventilation and tachypnea during respiratory failure and then to potentially prevent the risk of P-SILI.

## Data Availability

Data are available upon reasonable request to the corresponding author.
